# Experiences With Safer Conception Services for HIV-Serodiscordant Couples at a Referral Hospital in Nairobi, Kenya

**DOI:** 10.3389/frph.2021.693429

**Published:** 2021-11-04

**Authors:** Anne Kaggiah, Grace Kimemia, Hellen Moraa, Peter Muiruri, John Kinuthia, Alison C. Roxby

**Affiliations:** ^1^Department of Research and Programs, Kenyatta National Hospital, Nairobi, Kenya; ^2^African Population and Health Research Center, Nairobi, Kenya; ^3^Pediatrics Department, University of Nairobi, Nairobi, Kenya; ^4^CoEHM Project, Kenyatta National Hospital, Nairobi, Kenya; ^5^Departments of Medicine and Global Health, University of Washington, Seattle, WA, United States

**Keywords:** HIV, serodiscordant couple, safer conception, HIV viral load, pregnancy, Kenya

## Abstract

Human immunodeficiency virus-serodiscordant couples are an important source of new HIV infections in Africa. When trying to conceive, uninfected partners may be at high risk of infection if the infected partner is not virally suppressed. Multiple strategies targeting safer conception exist, but these services are limited. However, when services are available and used, serodiscordant couples can be protected from HIV transmission, and safe to have children if desired. To successfully introduce, integrate, promote, and optimize the service delivery of safer conception with HIV care, it is crucial to understand how HIV-serodiscordant couples perceive and experience these services. Further, viral load monitoring can be critical to safer conception, but there is limited literature on how it informs the decision of the partners about conception. This qualitative study describes the knowledge, perceptions, and experiences of both safer conception services and viral load monitoring among 26 HIV-serodiscordant couples seeking safer conception care at a referral hospital in Nairobi, Kenya. In-depth interviews of HIV-serodiscordant couples were conducted from April to July 2017, and transcripts were analyzed to identify the themes central to the experience of safer conception services of couples and viral load monitoring. Serodiscordant couples reported success in using some of the safer conception methods and had positive experiences with healthcare providers. However, despite using the services, some were concerned about HIV transmission to the seronegative partner and baby, while others faced challenges when using pre-exposure prophylaxis (PrEP) and vaginal insemination. Overall, their motivation to have children overcame their concern about HIV transmission, and they welcomed discussions on risk reduction. Moreover, supportive clinic staff was identified as key to facilitating trust in safer conception methods. Furthermore, viral load monitoring was identified as integral to safer conception methods, an emerging theme that requires further evaluation, especially where routine viral load monitoring is not performed. In conclusion, healthcare providers offering safer conception services should build trust with couples, and recognize the need for continual couple counseling to encourage the adoption of safer conception services.

## Introduction

Human immunodeficiency virus-serodiscordant couples are estimated to represent 2–8% of the HIV-affected couples in Africa and 4.8% in Kenya (1, 2), and are an important source of new HIV infections in Africa (3, 4). Serodiscordant couples often desire fertility, leading them to practice unprotected sex where the uninfected partner may be at high risk of HIV infection if the infected partner is not virally suppressed (5, 6). In the era of undetectable = untransmittable (U=U), it is important to note that viral load monitoring is not always available in a timely manner to these couples. Fortunately, multiple strategies for safer conception exist (7). These include vaginal insemination (4), male circumcisions, *in-vitro* fertilization (IVF) (4), sperm washing (4, 8), antiretroviral therapy (ART) for viral suppression of the seropositive partner (4, 8), pre-exposure prophylaxis (PrEP) for the seronegative partner to prevent HIV transmission, timed unprotected intercourse (4, 8, 9), and the diagnosis and treatment of sexually transmitted infections (STIs) (7). Serodiscordant couples who were offered and used safer conception services felt safe and protected from HIV transmission and empowered to have children (4, 10). Evidence shows that HIV-serodiscordant couples are able to discuss and use available safer conception services (4, 11).

However, the uptake of safer conception services is affected by various concerns. These include the integrity of sperm during home vaginal insemination, conflicting religious beliefs (4, 12), costs associated with sperm washing and IVF (3, 4, 12), difficulties in identifying the fertile period, perceived risk of HIV transmission during timed unprotected intercourse (4), poor adherence or perceived adverse events regarding ART or PrEP use, and beliefs about condoms as the primary prevention for HIV transmission (4, 13). While couples are eager to learn from healthcare providers about safer conception services (8, 10), the lack of support (8, 11), perceived judgment (8, 14), and stigma (13, 14) from providers can result in the reluctance of couples to initiate conversations of fertility desires and seek safer conception services (3, 8).

To successfully introduce, sustain integration, and optimize service delivery of safer conception services with HIV care it is crucial to understand how HIV-serodiscordant couples perceive and experience these services (15). Further, there is limited literature on how viral load results inform the decision of partners to conceive yet HIV viral load monitoring can be a critical part of safer conception. This qualitative study aimed to describe the knowledge, perceptions, and experiences of both safer conception services and viral load monitoring among HIV-serodiscordant couples seeking care at a referral hospital in Nairobi, Kenya.

## Methodology

### Study Design, Population, and Setting

Serodiscordant couples were eligible if they expressed fertility desire and were receiving HIV care at the Couple Counseling Center, Comprehensive Care Clinic, and Reproductive health clinics at the Kenyatta National Hospital (KNH) in Nairobi, the largest national referral and teaching hospital offering specialized health care services. Accordingly, we conducted qualitative in-depth interviews of couples from April to July 2017 to explore the knowledge, perceptions, and experiences of couples with safer conception services and viral load monitoring.

### Recruitment and Consenting Procedures

Site sensitization was conducted at the Couple Counseling Center, Comprehensive Care Center, and Reproductive health clinic in the KNH by the study team. The healthcare workers providing care to HIV-serodiscordant couples were informed about the study and requested to refer serodiscordant couples who wanted to conceive and were receiving safer conception services for study participation. Serodiscordant couples presenting in these clinics were recruited if they wanted to conceive and were receiving safer conception services. In addition, both members of the couple should have been available and willing to provide written informed consent.

Five couples receiving safer conception services were recruited and data analyzed, additional couples who had received at least two sessions of safer conception counseling, where the female partner was within the fertile age and had one or no children, were identified and invited to participate. These factors were considered because couples attending multiple counseling sessions were a rich source of information (16), while the age and number of children influence decisions about conception among HIV-serodiscordant couples (10, 17, 18).

Those willing to participate were informed about the study objectives and procedures and written informed consent was obtained from all the participants prior to study participation. If a couple was found ineligible, refused to participate, or was unavailable, the study staff would approach a subsequent couple from the same recruitment site. All interviews were conducted in a private room, and all study participants were reimbursed Ksh.500 ($5) for their time and transport expenses.

### Development of Interview Guides

Employing the grounded-theory methodology, we did not define *a priori* hypotheses. A structured interviewer guide inquiring about sociodemographic characteristics was utilized. The first section asked participants with fertility desire about their knowledge, perceptions, and experiences with safer conception services, whilst the second assessed their knowledge, perceptions, and experiences with HIV viral load monitoring as part of these services. These questions were developed from previous studies conducted among HIV-serodiscordant couples seeking safer conception services (19), and refined after pilot testing.

### Data Collection

Face-to-face audio-recorded semi-structured in-depth interviews were conducted by experienced social scientists trained on the protocol for 1 week prior to data collection. The training included a review of the semi-structured interview guides, informed consent, and qualitative data collection procedures. The in-depth interviews were conducted in the language preferred by the participants, either English or Kiswahili. The interviews focused on the knowledge, perceptions, and experiences with safer conception services and viral load monitoring. To better understand their knowledge, perceptions, and experiences, the HIV-serodiscordant couples were separated for individual in-depth interviews. This was to avoid the effect of the response of one partner on the other and prevent the dominance of the opinion of an individual.

### Data Analysis

The audio-recorded interviews were translated into English and transcribed. All the transcripts were independently reviewed and coded by three investigators, using a constant comparative approach (20, 21).

The data from the initial participants that were recruited was first analyzed and coded. These codes subsequently guided additional data collection and analysis. New data was constantly compared with the previous data for consistencies and differences. Additionally, emerging themes from the transcript of an individual were compared to that of their partner for consistency or variance and subsequently grouped into categories for research team discussion to ensure validity. To check for the consistency of text interpretation, coding was compared across the coders using an agreed-upon codebook. Those with discrepancies were discussed by the research team until resolution. After all the interviews were coded, the dominant themes were organized and representative quotes were chosen to illustrate the themes in the words of the participants. Two coders (AK and HM) met weekly to discuss the emerging themes, and codes applied. Any differences in coding were discussed with the third coder (GK), in consultation with other members of the investigating team (JK and AR), until consensus was achieved. DEDOOSE Software Version 8.0.35, was used for data management and organization (22).

The study was approved by the Kenyatta National Hospital/University of Nairobi Ethical Research Committee (KNH/UON ERC) (Ref: P4/01/2017) and the University of Washington Institutional Review Board (Ref: STUDY00000953).

## Results

A total of 31 couples were approached to participate, of whom 4 couples declined to participate, while one couple was not eligible because they were not fluent in English or Kiswahili. New topics ceased to emerge after 26 couples were interviewed, and the data saturation was deemed to have been reached (23). The mean age among the members of the HIV serodiscordant couples was 39 (23–43) and 35 (23–56) years, for males and females, respectively. Slightly more women than men had more than 8 years of formal education (21, 40.3 vs. 18, 34.6%). The average duration of partnership was 5.4 years, and the average number of children was 1. There were more female (18, 69.2%) than male partners (8, 30.2%0) who were HIV-seropositive. Pre-exposure prophylaxis (8, 30.8%) was the most commonly used method of safer conception, while sperm wash (1, 3.8%) was the least used. However, while few couples used both PrEP and ART (2, 7.7%) as methods of safer conception, others have not used (4, 15.4%) the method. The majority (17, 69.2%) of couples were recruited from the Couple Counseling Center, while the least (3, 11.5%) were recruited from the Reproductive Clinic (Table 1).

**Table 1:** Baseline characteristics of the study sample (*N* = 26 HIV-serodiscordant couples).

**Sociodemographic characteristics**	**Mean or *n* (%)**
**Age in years**Male Female	39 (23–56) 35 (23–43)
**Education** **>** **8 years**Male Female	18 (34.6) 21 (40.3)
Number of years in partnership	5.4
Number of children	1
**HIV-seropositive partners**Male Female	8 (30.8) 18 (69.2)
**Method of safer conception**ART PrEP Vaginal Insemination Timed unprotected intercourse ART and PrEP Sperm wash None	4 (15.4) 8 (30.8) 4 (15.4) 3 (11.5) 2 (7.7) 1 (3.8) 4 (15.4)
**Recruitment clinics**Couple counseling center HIV Comprehensive care center Reproductive Clinic	17 (65.4) 6 (23.1) 3 (11.5)

Several key themes related to the experiences of HIV-serodiscordant couples with safer conception methods and HIV viral load monitoring emerged from this study. An important theme was that couples had often been discouraged from attempting pregnancy at other sites, and were relieved to find a clinic that was interested in assisting them to achieve safe pregnancy. Overall, the couples described positive experiences with the services provided and the staff. Other notable themes included the challenges of accessing specialist fertility services, difficulties in adhering to some of the safer conception protocols, concerns about the effectiveness of PrEP, and continuing misinterpretation of viral load test results. Though the couples were interviewed separately, gendered differences in the experience of receiving safer conception services were not found, and couples greatly appreciated that these services were offered to them as a unit. The differences found were based on the serostatus of the partner, with seronegative partners expressing less knowledge about HIV and viral load testing. To fully describe the experiences of couples in our study, we report the key themes here for each step of the safer conception process.

### Referral for Care Was Welcomed by Couples Seeking Fertility

Some partners from other clinics within and without KNH reported that they were referred for safer conception services when these services and expertise were perceived as unavailable during their routine care. The Comprehensive Care Center within KNH offers routine HIV services such as the provision of ART, psychosocial support, and counseling services.

Based on the description of the participant, the Comprehensive Care Center offered limited counseling services and support to serodiscordant couples with fertility desires. Outside KNH, the health facilities that offer HIV services had little or no experience in supporting HIV-serodiscordant couples with fertility desire.

Couples who expressed fertility desire were mostly referred to the Couple Counseling Center at KNH, as described by an HIV-seronegative female partner who sought services at Comprehensive Care Center at KNH:

“*Yes, she was going there [Comprehensive Care Center]…, then we were advised to come to this clinic [Couple Counseling Center] so that we can get the right assistance…they said this [Couple counseling Center] was the right place for the two of us because there are things that are accommodated here [Couple counseling center]…*” Couple 13 HIV-seronegative male partner

The couples were grateful for the option of a specialty clinic focusing on their fertility concerns, enabling them to access an expert understanding of HIV and its implications.

### Couples Had Prior Negative Experiences With Community Providers and More Positive Experiences With Specialized Safer Conception Services

During the routine clinic visits, the couples would talk to their health providers about their fertility desires. This topic was met by mixed reactions from the providers who either showed empathy or outright displeasure toward the couple due to the perceived risk assessment of their health providers. There was a perception that HIV-serodiscordant couples should not have children because of possible HIV transmission to the seronegative partner and unborn child. In such instances, the participants reported that the health care providers from other facilities discouraged them from having children:

“*.she (healthcare provider) told us that getting a second child is just risking, so she made us scared and we knew there was no way of getting a baby*.” Couple 11 HIV-seronegative male partner

“…*She was afraid and she said that according to how the doctor tested her, he [the doctor] told her that she cannot have a baby*.” Couple 9 HIV-seronegative male partner

In addition, the HIV-serodiscordant couples were worried about conceiving naturally with their HIV status. When the couples were referred to the Couple Counseling Center, they learned about safer conception services, rejuvenating their fertility desires, as well as their hope and motivation toward having seronegative children, and maintaining their serodiscordant relationships:

“*We had not tried (to have a baby). In fact, we had abstained from intercourse for a year…Because of that state of…one person has HIV and the other does not… We came here [Couple Counseling Center] for counseling, their services were good, they counseled us, and we saw that since we can get another child, we can stay as a couple*.” Couple 2 HIV-seropositive female partner

“*They [safer conception services] are very important because they bring hope and light to some couples because at least now you know that I can still have a baby naturally*.” Couple 16 HIV-seronegative female partner

Further, the couples who accessed safer conception services felt that the services were important for the protection of the seronegative partner and unborn child from HIV transmission. The benefits of the services were highlighted by couples during the in-depth interviews.

“*Yes, they [safer conception services] are beneficial because ….one partner is positive and the other one is negative. In order to protect the other one from being infected we need to use the safer methods. This is for the future of us and of the child that will be born*.” Couple 9 HIV-seronegative male partner

“*The services [safer conception] offered are not bad rather they are helping us. They help protect our partners who are HIV negative from being infected. If they are on medication and we have intercourse normally, using the medicine they have already protected themselve*s.” Couple 5 HIV-seropositive female partner

The couples who visited the Couple Counseling Center encountered health providers that are knowledgeable about safer conception services. The couples reported that it was easy to speak about their fertility intentions where healthcare providers reacted positively to their concerns. Moreover, the participants appreciated the services offered as described by the following excerpts:

“…*…The ones providing the services here are good-hearted compared to other places I have been. When I come here [Couple Counseling Center] I always feel at home. I respect this place*.” Couple 9 HIV-seronegative male partner

“*If you want to get a baby it is not a must you struggle, you just come….and inform the doctors that you want to conceive………They [healthcare providers] received us well……*” Couple 25 HIV-seropositive female partner

For many partners, coming to the safer conception clinic was the conclusion of a multistep journey toward fertility, including coming to terms with their serodiscordant status, learning to hope that fertility would be possible, seeking correct expertise to guide their fertility journey, and self-education to understand and weigh competing options. The couples were grateful for the positive experiences of the safer conception clinic, despite carrying the emotional weight of the journey to arrive at this place of support and hope.

### Couples Described High Levels of Knowledge of Safer Conception Services

While conducting in-depth interviews with different partners, we noted that the female participants were more knowledgeable than the male participants about safer conception methods such as timed unprotected intercourse, use of PrEP, vaginal insemination, and use of ARVs. The female partners provided detailed descriptions of the safer conception methods as informed by healthcare providers. Table 2 above shows some of the methods partners were knowledgeable about:

**Table 2:** Excerpts describing couples' knowledge of safer conception methods.

**Method**	**Quote**
Timed unprotected intercourse	“*We started with my cycle, …we had to go through and see the most proper… date that I could conceive….I was actually told that from the 10th day that is probably the time when I am fertile……that is the time we should have sex without protection*.” Couple 24 HIV-seropositive female partner
PrEP	“…. *We were told that PrEP is of help because we have used [it] for 3 months and he has come for the test and he is negative….It helps the person who is HIV-seronegative not to be infected……*.” Couple 22 HIV-seropositive female partner
Vaginal insemination	…*…*.“*Ejaculate in the condom and ……remove it and……. deposit [in the vagina] it [semen] using the syringe…*.” Couple 22 HIV-seronegative male partner
ARVs	“*They told us of the methods of how we can do it….My partner was introduced to ARVs, he was not taking them before because the CD4 count was still ok, but he was told now he has to start using them…. his viral load was checked and we were told it is ok and we were just given a go ahead to just try….without the condom, to try you count the safer days and you just try naturally*.” Couple 10 HIV-seronegative female partner

The knowledge of these methods was very specialized, as none of them are commonly used in Kenya and therefore showed the investment of the couples in the process of pursuing safer conception services, as well as demonstrating that the clinical education process was effective at improving knowledge of different safer conception options.

### Couples Reported Diverse Sources of Information

The sources of information about safer conception methods varied based on the exposure to different health facilities and mass media. One of the participants reported learning about safer conception services from health care providers during routine visits at the Couple Counseling Center or while attending support groups organized by the center.

“…*We had a meeting on Saturday [at the clinic].The (safer conception) methods were explained in that meeting…*.” Couple 5 HIV-seropositive female partner

“*When my wife heard that I was using drugs, she … wondered what the drugs [were] for… I told her they [were] for prevention. She asked about what am preventing [HIV transmission]….so today we came to the clinic together……and she was told that they are for prevention because I am positive and she is negative. So she heard that from doctor and that was affirmed.”* Couple 23 HIV-seropositive male partner

In addition to the use of ARVs by the HIV-seropositive partner as a method of safer conception, some partners heard about PrEP from televised mass media during its launch by the Kenyan Government. In other cases, as narrated by an HIV-seropositive female partner during an in-depth interview, the participants would explore the internet for information on how to protect the negative partner:

“*On television, is it the one [PrEP] that was being launched just the other day…. I just heard it; it protects you from getting infected….the negative partner*.” Couple 6 HIV-seronegative male partner

“…*.It is Google that told me that there is a drug [PrEP] that I can use that can protect the negative partner*.” Couple 14 HIV-seropositive female partner

Furthermore, some participants received information from friends or peers, to whom they have disclosed their HIV status. In one of the interviews, a participant reported that a friend, concerned about her not having another child, informed her about how she could safely conceive.

“…*.From a man who questioned me on why I had stayed for long without getting another [child]…….The man explained to me that there was a method of putting the sperms in the condom and I would be injected in the womb and I would conceive*.” Couple 5 HIV-seropositive female partner

Overall these experiences demonstrated that people sought information from diverse sources, and found it hard to determine the reliability of some of the information. The clinic provided a place to query experts, evaluate diverse information and make an informed decision about fertility and safer conception.

### Successes and Challenges With Using Some Safer Conception Methods

After receiving detailed information about the available safer conception methods from healthcare providers, some participants reported success in using some such as PrEP, and reported they felt safe and protected from HIV transmission:

“…*.My husband will be using those medications (PrEP) that will protect him from getting HIV, I feel safe*.” Couple 3 HIV-seropositive female partner

“…*. I know… the PrEP that I was given…. protects me*.” Couple 12 HIV-seronegative male partner

“…*….So as he uses that drug [PrEP], as we meet without condoms there is no way he can be [infected]…………*” Couple 12 HIV-seropositive female partner

However, one couple reported that adherence and adverse events made it difficult to take PrEP, though they understood the benefits of using PrEP to reduce HIV transmission to the negative partner.

“*Taking medication [PrEP] every day, to some of us is so tricky. I wish it could be for a shorter period, if at all you take and conceive…*.” Couple 16 HIV-seronegative female partner

“…*.with PrEP my partner was always complaining because of the reaction of the drugs*.” Couple 16 HIV-seropositive male partner

Though vaginal insemination was a preferred safer conception method among couples with an HIV-seronegative male partner, several couples reported it to be challenging due to the processes involved. Some female partners intimated that they found semen insertion *via* a syringe uncomfortable, while others doubted the effectiveness of the method because of semen spillage, and others found touching the genitalia uncomfortable:

“*The syringe method……it was a challenge, it was really a big challenge because… from school I learnt that sperms only survive outside for…I don't know how many hours. So, I was like… is it really going to work, you ejaculate then you put it in the syringe. It was a process, it is challenging*.” Couple 6 HIV-seropositive female

“…*like ejaculating in the condom and then you remove it and you deposit [semen] using the syringe… I didn't find it more effective for me, because when you are doing all that, you will lose some [semen]…Then putting [semen into the vagina] then… Waiting…an hour or 30 minutes*.” Couple 21 HIV-seronegative female partner

Furthermore, some participants expressed concerns about sperm washing, citing specialist providers who they felt were indifferent or unprofessional. Others were concerned that the method was ineffective and costly:

“*It is quite a lot of money for consultation and….inserting the washed sperms… that process of taking the sperms you go to the clinic in town you are told to wait… I found that unprofessional, I said I wouldn't want to go through it*.” Couple 10 HIV-seropositive male partner

“…*.For sperm wash you must be financially stable, and it is not 100% guarantee that it will work, if it fails that means you are going to repeat again…*” Couple 16 HIV-seropositive male partner

In general, having so many methods of safer conception was perceived by couples to be another challenge to navigate and another barrier to having a baby. While some were successful, overall, the process of safer conception was felt to be more complicated than natural conception and required a significant investment of time and energy.

### Perceptions of Safer Conception

The study participants had varied perceptions of the different types of safer conception methods. During the interviews, some participants expressed that vaginal insemination was artificial and the sperms would not be viable:

“*If you take the syringe out of the condom you never know if ……… the sperms are already dead because of the air*.” Couple 6 HIV-seronegative male partner

“*Artificial…….That is just what I think because just like in cows, they go to the veterinary and they conceive*.” Couple 8 HIV-seropositive male partner

Further, couples explained how they tracked their menstrual periods to identify fertile days, noted as important especially if the couples chose timed unprotected sex. However, based on the interviews, the couples displayed minimal understanding on how to count days and identify fertile days:

“…*… I am beginning my periods today, so from today I shall count 8 days, during those days we can have sex, after 8 days we have to skip 3 days and we meet on the 4 day and we alternate 4 times for 4 days the alternation is to make the sperms be strong*.” Couple 12 HIV-seropositive female partner

“*You know when women have had their periods after that the chances of getting a child are high or slightly before, so you should [have intercourse] in those 3 days continually*.” Couple 11 HIV-seronegative male partner

This theme showed that couples took the need to acquire specialized knowledge seriously on their path to fertility.

### Concern for HIV Transmission Despite Safer Conception Methods

Although the concern for HIV transmission was the greatest motivator for clinic attendance, some participants expressed their ongoing concern about HIV infection despite being offered safer conception methods. For instance, in one couple, the HIV-seronegative male partner doubted the effectiveness of PrEP and the low viral load of his HIV-seropositive partner.

“*The hardest part was, are these drugs…. [PrEP] effective? Suppose they don't [work], whom do you blame, do you blame these professionals or do you blame your wife?*” Couple 22 HIV-seronegative male partner

“*He cannot accept even when we were being told that for example if my viral load is low you can risk having sex without the condom because it is not easy…. to infect him, he said never*.” Couple 22 HIV-seropositive female partner

This theme underlined the fear of HIV acquisition and transmission that was a great barrier to overcome to achieve safer conception. Despite the counseling and training that couples had received during safer conception services, the fear of HIV remained. In addition, some seropositive partners seemed more concerned about HIV transmission than their seronegative partners.

“*I………heard……. we can stay together without him being [infected]. I was very happy because I……did [not] want to infect him*.” Couple 12 HIV-seropositive female partner

“*I am [on] medication [and] she is not [on] medication….[when] we have [unprotected] sex….she is going to get [HIV infection]………*” Couple 16 HIV-seropositive male partner

On the other hand, the seronegative partners were supportive and motivated to get a baby using these services.

“……*She was found to be [HIV] positive and I was [HIV] negative…….The doctor asked [us] how shall [we live]?………I told him that I …[will not] abandon her just because she is [HIV] positive*.” Couple 12 HIV-seronegative male partner

“*[Safer conception services]….are very important because they bring hope and light to….couples ………I can still have a baby naturally…….”* Couple 16 HIV-seronegative female partner

These themes emphasize the importance of providing these services to couples rather than individual members of the couple so that concerns of HIV transmission can be addressed and spousal support can be encouraged.

### Understanding of Viral Load as a Measure of Infectivity and a Means to Reduce HIV Transmission

The couples reported that, prior to discussing safer conception methods, healthcare providers required the viral load testing of the HIV-seropositive partner. In addition, they understood that high viral loads level would deter or derail their plans to have children:

“*The first requirement was a viral load…so when my viral load was below 20 copies, they [healthcare provider] gave me a green light to go ahead.”* Couple 1 HIV-seropositive female partner

“*Yes, it is important, because during our meetings with [healthcare provider] he says that when the viral load is undetectable that is the right time to conceive. But when it's high it is risky to conceive*.” Couple 8 HIV-seropositive male partner

Some partners in serodiscordant relationships demonstrated knowledge about viral load, and understood that a low or undetectable viral load in the seropositive partner meant that HIV transmission to the seronegative partner and baby would be reduced:

“*When the viral load is high the chances of infecting your partner are high unlike when it is low or undetectable.” Couple*.” Couple 8 HIV-seropositive male partner

“…*If your viral load is low, you can't infect your partner*.” Couple 9 HIV-seropositive female partner

In this case, the couple explored the use of ARVs to reduce the viral load of the HIV-seropositive partner to undetectable levels. This could only be achieved if the seropositive partner adhered to their ART medication:

“*It is the measuring of the amount of virus in your body. It checks if the drugs are working, and if the amount of virus is reducing or they are multiplying*.” Couple 3 HIV-seropositive female partner

“…*Viral load is like when she doesn't use her medication well so the HIV virus goes on higher levels, but when she does take her medication properly the virus goes completely down*.” Couple 7 HIV-seronegative male partner

Some seronegative partners lacked knowledge of the viral load test prior to attending the Couple Counseling Center, attributed to the fact that most of the HIV-seropositive partners would attend the HIV clinic alone for their routine checkups, medication, and viral load testing. However, both partners were expected to attend safer conception counseling where HIV-seronegative partners learned about viral load:

“*I have not heard about such a thing [viral load]…. I heard her asking the doctor that she wanted to know her levels because I want us to have the baby…The doctor…checked and told her she is negative something… and told her that she can conceive because her health is good. …. I did not understand*.” Couple 9 HIV-seronegative male partner

“*I have never heard [of viral load] and I don't know if he has ever been tested, I don't know*….” Couple 21 HIV-seronegative female partner

In addition, a few participants did not understand how to use viral load monitoring as a method of safer conception. As described by one, she thought that she should try conceiving when the viral load was high.

“……*They [healthcare providers] have to determine if I was able to get a baby because my status could be that the viral load is low and may not be able to carry the pregnancy……….they [healthcare providers] will try to take it [viral load] high for me to be able to get pregnant…*..” Couple 12 HIV-seropositive female partner

Overall, the couples benefited from understanding the role of the HIV viral load as part of the path toward safer conception. However, it remained a concept that was better understood by the HIV-positive partners, and viral load testing was understood as a potential barrier or possible delay to fertility plans.

## Discussion

This qualitative research elicited the viewpoints of HIV-serodiscordant couples seeking safer conception. Despite the sensitive topic, the couples shared their challenges seeking safer conception services while protecting the seronegative partner.

Overall, the themes identified reinforced that safer conception services were appreciated by the discordant couples, and resulted in couples with knowledge of their fertility options, good comprehension of the methods to reduce risk as they pursued fertility, and a reasonable understanding of the role of viral load suppression in safer conception. While some safer conception methods were unpopular, such as sperm washing and self-insemination, others appeared acceptable and couples displayed a nuanced understanding of risks and benefits. Finally, couples appreciated safer conception education and counseling services at the specialty clinic.

This research showed that couples relied on healthcare workers and support groups as main sources of information and media, the internet, and friends as other sources. This underscores the need for specialized or integrated clinics with providers who have specific training on safer conception methods, as well as integration of this training into clinics where couples seek HIV care. Despite the overall broad knowledge demonstrated by couples, we did identify some couples with incorrect or incomplete knowledge of some of the safer conceptions methods, as seen in other studies (24). Continuous counseling of couples about safer conception may be needed to reinforce novel concepts, especially for those with limited health literacy.

While we were pleased with the satisfaction expressed by couples regarding services at the safer conception clinic, there is likely a much greater need from outside the referral center, as many were unaware of the existence of these services before referral by a provider. Another study conducted among serodiscordant couples in Kenya noted missed opportunities to provide safer conception, which could be improved by integrating services and training all healthcare workers providing care to these couples (24).

Given the very real concerns expressed by couples about HIV transmission to their partner or infant, there is a need for comprehensive and trusted services targeting this very important life choice. Counseling couples seeking safer conception services on a regular basis can help alleviate such concerns, making these services acceptable to these couples (14). Viral load monitoring was required prior to offering safer conception methods, however, some seronegative partners had limited understanding of the implications of viral load testing results or were unaware of the viral suppression states of their partners. There is a need to incorporate seronegative partners during ART adherence counseling of the seropositive partners to achieve undetectable viral load. Programs should understand that couples seek expert advice and need reassurance from professionals about their choices given that there is a risk to their closest loved ones.

Some methods, particularly vaginal insemination and sperm washing, were unpopular among couples due to their complexity and expense. While PrEP may supplant these methods, the couples also reported mixed opinions about PrEP, with seronegative persons expressing unwillingness to take daily pills. Current safer conception practices have evolved to rely more heavily on PrEP as a safer conception method. However, this research showed that even when HIV-serodiscordant couples are motivated by fertility desire, they may still be unwilling to use PrEP.

Despite U=U dominating the discourse around HIV risk, some have asked if clinics such as this continue to be necessary. Our research shows that HIV-serodiscordant couples face many barriers to conception, and fear infecting their loved ones. Most participants felt that these services were important for serodiscordant couples because they motivated, gave hope, and kept them together as they sought children. In addition, motivated couples are likely to accept these services despite the challenges in using some of the methods. Thus, these services need to be integrated with HIV care, and healthcare providers need training so that they can initiate conversations on safer conception with HIV-serodiscordant couples expressing fertility desire (25).

As viral load testing becomes easier and more convenient in sub-Saharan Africa, our findings show that HIV-serodiscordant couples are able to integrate this knowledge into safer conception. Most couples were aware that HIV viral load testing for the seropositive partner was essential, and couples had good knowledge of reduced HIV transmission from a person with undetectable viral load, and acknowledge the need for the seropositive partner to be adherent to their ARV medication. However, we did identify couples who lacked knowledge on viral load testing, especially seronegative partners who may neither attend HIV clinics nor receive the information. It remains, then, a key tenet of safer conception care to ensure that prior to attempting conception, both members of the couple understand the role of viral load in HIV transmission and the importance of ART adherence and viral load suppression.

### Strengths and Limitations

To our knowledge, this is the first study within a public clinic setting offering safer conception services in Kenya, as most studies have been conducted in research settings. In addition, there is limited research on viral load monitoring as part of safer conception services, and the findings from this study could be used to bridge some of the gaps in the literature. A limitation is that the research was conducted in an urban setting, which may not be representative of the experiences of couples in rural areas. The members of the couple were interviewed separately to encourage the perspective of each member; however, with this approach, we were not able to observe the couple dynamics. This research was conducted when both PrEP and viral load monitoring were relatively new in Kenya; therefore current perspectives may be different as these technologies are more widely adopted.

### Conclusions

This qualitative study showed that the couples had positive experiences with safer conception, received counseling and education, and were motivated to attempt conception despite their discordant status. The couples endorsed safer conception services delivered by healthcare providers with positive attitudes toward the fertility intention of serodiscordant couples. Interviews revealed that serodiscordant couples remained concerned about HIV transmission, and faced challenges when using some safer conception methods. In addition, viral load monitoring was required prior to offering safer conception methods, an emerging theme that needs to be evaluated further in areas where routine viral load monitoring is not performed. Overall, their motivation to have children helped them overcome challenges and remain open to discussions on risk reduction. Finally, HIV-serodiscordant couples were enthusiastic about ART and PrEP as a way to protect seronegative partners and appreciated counseling and reassurance regarding unprotected sex in that setting. Providers should consider these needs when offering safer conception services, and consider that ongoing couple counseling may be needed to alleviate concerns and challenges, thus making these services acceptable.

## Data Availability Statement

The raw data supporting the conclusions of this article will be made available by the authors, without undue reservation.

## Ethics Statement

The studies involving human participants were reviewed and approved by Kenyatta National Hospital/University of Nairobi Ethical Research Committee (KNH/UON ERC) (Ref: P4/01/2017) and the University of Washington Institutional Review Board (Ref: STUDY00000953). The patients/participants provided their written informed consent to participate in this study.

## Author Contributions

AK contributed to the conceptualization, data curation, funding acquisition, investigation, methodology, reviewing, and editing of the manuscript. AR contributed to the conceptualization, data curation, funding acquisition, investigation, methodology, supervision, resources, reviewing, and editing of the manuscript. GK contributed to the methodology, data validation, writing original draft, reviewing, and editing the manuscript. HM contributed to the methodology, investigation, data validation, reviewing, and editing of the manuscript. JK contributed to the conceptualization, funding acquisition, investigation, methodology, supervision, resources, reviewing, and editing of the manuscript. PM contributed to the methodology, resources, reviewing, and editing of the manuscript. All authors contributed to the article and approved the submitted version.

## Funding

This research was funded by the National Institute of Health Research Training Grant #R25TW009345 funded by the Fogarty International Center, the NIH Office of the Director Office of AIDS Research, the NIH Office of the Director Office of Research on Women's Health, the National Heart, Lung and Blood Institute, the National Institute of Mental Health, and the National Institute of General Medical Sciences. AR was supported by NICHD K23HD071788 and NIH-supported Centers for AIDS Research: AI027757.

## Conflict of Interest

The authors declare that the research was conducted in the absence of any commercial or financial relationships that could be construed as a potential conflict of interest.

## Publisher's Note

All claims expressed in this article are solely those of the authors and do not necessarily represent those of their affiliated organizations, or those of the publisher, the editors and the reviewers. Any product that may be evaluated in this article, or claim that may be made by its manufacturer, is not guaranteed or endorsed by the publisher.
